# Using the T Cell Receptor as a Biomarker in Type 1 Diabetes

**DOI:** 10.3389/fimmu.2021.777788

**Published:** 2021-11-17

**Authors:** Maki Nakayama, Aaron W. Michels

**Affiliations:** ^1^ Barbara Davis Center for Childhood Diabetes, University of Colorado School of Medicine, Aurora, CO, United States; ^2^ Department of Pediatrics, University of Colorado School of Medicine, Aurora, CO, United States; ^3^ Department of Immunology and Microbiology, University of Colorado School of Medicine, Aurora, CO, United States; ^4^ Department of Medicine, University of Colorado School of Medicine, Aurora, CO, United States

**Keywords:** T cells, TCR sequencing, autoimmunity, type 1 diabetes, HLA, MHC

## Abstract

T cell receptors (TCRs) are unique markers that define antigen specificity for a given T cell. With the evolution of sequencing and computational analysis technologies, TCRs are now prime candidates for the development of next-generation non-cell based T cell biomarkers, which provide a surrogate measure to assess the presence of antigen-specific T cells. Type 1 diabetes (T1D), the immune-mediated form of diabetes, is a prototypical organ specific autoimmune disease in which T cells play a pivotal role in targeting pancreatic insulin-producing beta cells. While the disease is now predictable by measuring autoantibodies in the peripheral blood directed to beta cell proteins, there is an urgent need to develop T cell markers that recapitulate T cell activity in the pancreas and can be a measure of disease activity. This review focuses on the potential and challenges of developing TCR biomarkers for T1D. We summarize current knowledge about TCR repertoires and clonotypes specific for T1D and discuss challenges that are unique for autoimmune diabetes. Ultimately, the integration of large TCR datasets produced from individuals with and without T1D along with computational ‘big data’ analysis will facilitate the development of TCRs as potentially powerful biomarkers in the development of T1D.

## Introduction

A T cell receptor (TCR) determines antigen specificity of T cells by interacting with a peptide-major histocompatibility complex (peptide-MHC), and signals received through the TCR along with the CD3 complex are the primary components that regulate function and fate of T cells. Individual T cells express unique TCRs, and therefore TCR sequences can be used as an identifier of T cells that are specific to particular antigens and involved in immune responses. In this review, we will focus on the potential use of TCR sequences as non-cell based T cell biomarkers for type 1 diabetes (T1D), a tissue-specific autoimmune disease targeting insulin-secreting pancreatic beta cells (1–3).

Several features of self-reactive T cells make it challenging to develop T cell biomarkers in diabetes (4). First, the frequency of autoreactive T cells is extremely low in the peripheral blood, estimated to be 1/10^5^ – 1/10^6^. Second, response to peptide-MHC by autoreactive T cells tends to be minimal compared to anti-cancer or anti-pathogen T cell responses (5, 6). Third, healthy individuals with T1D-risk MHC molecules can have autoreactive T cells that are quantitatively and functionally similar to those in T1D patients (7). TCR sequencing allows for the analysis of TCR clonotypes from tens of millions of T cells using nucleotide samples rather than living cell biospecimens and may overcome many of these challenges when appropriately utilized. Advantages provided by TCR biomarkers include (1) living T cells are not required for assays; (2) intra- and inter-assay variations due to cell conditions and operator performance are minimized; and (3) extremely infrequent and low-responding T cells are detectable by recently emerging high-throughput sequencing technologies. Here, we will review current knowledge about TCR repertoires and clonotypes specific for T1D and address the knowledge gaps to develop TCR biomarkers that can stratify individuals throughout the stages of T1D development.

## Tri-Molecular Complex Consisting of TCR, Peptide, and MHC Molecules

TCRs expressed by classical T cells are composed of alpha and beta chains, both of which are formed by somatic recombination of the variable (V) and joining (J) segment genes (and diversity [D] for beta chains). In humans, 45 TRAV and 52 TRAJ genes have been identified as functional V and J segment genes for alpha chains (8, 9). Likewise, there are 49 TRBV, 2 TRBD, and 13 TRBJ functional V, D, and J segment genes in the beta chain locus (10, 11). During maturation in the thymus, individual T cells undergo rearrangement of segment genes, resulting in one V, one D (for beta), and one J segment genes assembled adjacent to each other. Since additional nucleotides are often inserted or deleted between the segments, billions of junction sequences with hundreds of different V, D, J combinations are possibly assembled (12–14). Experimentally, each adult person is expected to have over 100 million TCR clonotypes uniquely expressed by hundreds of billions of individual T cells in the body (15–19). There are three regions, called complementarity determining regions (CDR), that directly interact with peptide-MHC complexes, thereby crucial to determine antigen specificity (20–22). Two CDR regions, CDR1 and CDR2, are included in the V segment, and the CDR3 region is formed at the junction between V, D (for beta), and J segments. Amino acid residues in the CDR3 regions closely interact with peptide, and thus are considered to be important to determine antigen specificity and are often used as a property of each TCR clonotype for TCR repertoire analysis.

## MHC Molecules in T1D

The major genetic determinant in susceptibility to most autoimmune diseases reside in the human MHC that contains the human leukocyte antigen (HLA) region. MHC molecules are heterodimers formed between alpha and beta chains that function to present peptides to TCRs on T cells. Class I molecules are on all nucleated cells and present antigens to CD8 T cells, while class II molecules are expressed by antigen presenting cells (e.g. B cells, dendritic cells, and macrophages) and present peptides to CD4 T cells. In T1D, specific HLA class I and II alleles are associated with increased risk (23, 24). Several HLA class I and II alleles confer risk for T1D and are associated with other autoimmune disorders (**Table 1**) (25, 26). DR is in close linkage disequilibrium with DQ such that the DR4-DQ8 and DR3-DQ2 haplotypes confer the greatest risk for T1D development. Both the alpha and beta chains of DQ molecules are polymorphic, and have the ability to form mixed molecules in cis and trans. As an example, the alpha chain of DQ2 can pair with the beta chain of DQ8 to form DQ8-trans (DQA1*05:01-DQB1*03:02) when both DQ2 and DQ8 are in the genotype. DQ8-trans has an odds ratio of disease development for T1D at 35 (35 times more likely to develop diabetes compared to those without these alleles), compared to odds ratios of ~11 and ~4 for DQ8 and DQ2, respectively (27, 28). Interestingly, HLA-DQ6 (DQA1*01:02-DQB1*06:02) provides dominant protection for T1D development with an odds ratio of only 0.03 (29, 30). The stark dichotomy of risk between DQ molecules highlights the important role of antigen presentation to TCRs in T1D.

**Table 1:** Common HLA alleles associated with type 1 diabetes risk.

Name	Allele	Associated Autoimmune Diseases
**HLA Class II**		
DQ8	DQA1*03:01-DQB1*03:02	Celiac disease, Addison’s disease
DQ2	DQA1*05:01-DQB1*02:01	Celiac disease, Addison’s disease
	DQA1*02:01-DQB1*02:02	Celiac disease, Addison’s disease
DQ8-trans	DQA1*05:01-DQB1*03:02	Celiac disease
DR4	DRB1*04:01	Rheumatoid Arthritis, Thyroid disease, Addison’s disease, Alopecia Areata
	DRB1*04:02	Rheumatoid Arthritis (protective), Thyroid disease, Addison’s disease
	DRB1*04:04	Rheumatoid Arthritis, Thyroid disease, Addison’s disease
	DRB1*04:05	Rheumatoid Arthritis, Thyroid disease, Addison’s disease
DR3	DRB1*03:01	Systemic Lupus Erythematous (SLE), Neuromyelitis Optica (NMO), Myasthenia Gravis, Thyroid disease, Addison’s disease
**HLA Class I**		
A2	A*02:01	Vitiligo
A24	A*24:02	unknown
B39	B*39:06	unknown
B18	B*18:01	unknown
B7	B*07:05	unknown

## Diversity of TCR Repertoires

Adults have approximately 10^8^-10^10^ unique TCR clonotypes (15, 17, 18, 31). With an assumption that the TCR repertoire size may represent a capacity for responding to diverse antigens, the TCR repertoire diversity in the blood has been examined to determine whether it is associated with immune conditions. For example, having diverse TCR repertoires is associated with desirable responses to immune therapies in cancer (32–34). In T1D, it has been reported that TCR repertoires in peripheral blood of T1D patients are less diverse compared with those without T1D (35). Thus, there may be trends of TCR repertoire sizes that are preferred by a certain immune condition. However, it should be noted that the diversity of TCR repertoires cannot specify a certain disease.

## Use of TCR Clonotypes as Surrogates to Quantify Antigen-Specific T Cells

TCR clonotypes determine antigen specificity, and therefore they can be utilized as a surrogate marker to evaluate the presence and prevalence of antigen-specific T cells in the blood. Frequencies of these antigen-specific TCR clonotypes can be quantified by high-throughput sequencing, which is expected to be more specific to individual diseases compared to surveying the broad TCR repertoire. Furthermore, once a panel of antigen-specific TCR clonotypes are determined, a single TCR sequencing assay allows for evaluating specificity to many antigens rather than needing to test specificity to each individual antigen. TCR sequencing has been done from different tissues in many disease states (36), including autoimmune disorders (37) and cancer (38–40). Remarkably, TCR sequencing has been shown to differentiate early-stage cancer patients from healthy individuals (41, 42). This strategy requires a list of TCR clonotypes beforehand that can be searched in blood samples, and such TCR clonotypes used as surrogate biomarkers need to satisfy three factors: (1) publicity (i.e. commonality and shared between individuals), (2) abundancy, and (3) disease specificity. Namely, T cells expressing the same or similar TCR clonotypes need to be commonly present in a number of people; frequency of such T cells in the blood of each person needs to be high enough for quantification; and presence or absence of such T cells needs to be associated with a disease state. In addition, with larger numbers of TCR clonotypes in a given panel, the more specific and sensitive an assay will become. Thus, identifying disease-specific TCR candidates is essential to establish a robust TCR sequencing assay that can discriminate a subset of individuals having a specific stage or feature of T1D such as those who have potential to respond to an interventional therapy.

There are several strategies to identify disease-specific TCR clonotypes. Since a significant portion of disease-specific TCRs are likely to recognize islet antigens, TCR clonotypes expressed by islet antigen-specific T cells are reasonable candidates for TCR biomarkers. Such T cell sources include peripheral blood T cells responding to islet antigen stimulation or enriched by staining with fluorescence-conjugated multimers consisting of an islet-derived peptide and a particular HLA molecule (43–45). Alternatively, TCR clonotypes identified in the target organ (i.e. pancreas or pancreatic islets) or draining lymph nodes may be also disease-specific. In any of these T cell sources, specificity (i.e. potential contamination of non-disease associated T cells) as well as sensitivity (i.e. missing a portion of antigen-specific T cells) needs to be carefully considered. For example, T cell samples enriched by antigen stimulation may contain only a few clonotypes that readily proliferate in response to the stimulation or could be non-specific T cells that proliferate due to “bystander effect.” Likewise, T cells in the pancreas and pancreatic lymph nodes may not necessarily be islet-reactive or disease-specific (46). On the other hand, T cell populations enriched by multimer staining may contain only those having high affinity to bind peptide-MHC complexes, and TCRs weakly binding to peptide-MHC may be missed. This possibility is likely important for autoreactive TCRs since T cell responsiveness to self-antigens tends to be low compared to pathogen T cell responses. Nevertheless, identifying TCR clonotypes from samples enriched with antigen-specific T cells is indispensable to identify disease-specific TCR candidates. These TCR clonotypes should then be assessed for frequency in peripheral blood of individuals with different stages of T1D to determine the ultimate association with disease status. The next subsections will summarize features of TCR clonotypes specific to islet-specific autoantigens as well as those potentially associated with T1D pathogenesis.

### Lessons From Islet-Specific TCRs in T1D Animal Models

Non-diabetic (NOD) mice spontaneously develop autoimmune diabetes and represent many features of human T1D including a T1D-susceptible MHC allele (I-A^g7^), homologous to HLA-DQ8, the development of insulin autoantibodies prior to diabetes onset, and insulitis. A number of T cell clones reacting with islet tissues have been isolated from pancreatic islets and spleens of NOD mice in the past few decades and further characterized for antigen specificity as well as TCR clonotypes (47). In the 1990’s, Santamaria and colleagues discovered that a large portion of CD8 T cells infiltrating NOD islets share an identical Valpha segment (i.e. TRAV16) along with a specific junction motif (i.e. MRD or MRE) (48), and subsequently identified a peptide derived from islet-specific glucose-6-phosphatase catalytic subunit-related protein (IGRP) as an epitope targeted by these CD8 T cells (49). Likewise, CD4 T cell clones as well as T-hybridoma cells that are reactive to insulin B-chain peptides have been established from NOD islets by a number of investigators using different methods (50–56). The majority of these T cells expresses TCRs containing specific Valpha and Jalpha segment motifs, TRAV5D-4 or TRAV10 along with TRAJ53 or TRAJ42. When mice are forced to have only T cells expressing TCRs containing TRAV5D-4, approximately one percent of CD4 T cells becomes specific to an insulin B chain 9-23 peptide (57), and the mice are susceptible to develop anti-insulin autoimmunity (58, 59). Alanine scanning and crystal structure analyses identified several amino acid residues in the TRAV5D-4 and TRAV10 CDR1 and CDR2 regions that are crucial to interact with the insulin peptide-MHC complex (59, 60). Also, among insulin B chain-specific CD4 T cells, those particularly recognize an insulin B chain 12-20 peptide prefer to express TCR beta chains containing a negatively charged amino acid (i.e. aspartic acid [D] or glutamic acid [E]) in the junction region (56). This observation is consistent with a notion that the I-A^g7^ T1D-susceptible MHC class II molecule, which has a positively charged patch in the surface area near the p9 pocket due to the lack of a negatively charged amino acid residue at the beta 57 position, engage TCRs having a negatively charged residue when p9 of peptides is not negatively charged (the position 20 of insulin B chain is glycine). Thus, these studies provide a molecular elucidation of how TCR motif selection occurs by interaction with a particular peptide-MHC complex.

T1D-specific TCR repertoires in rat models have been extensively studied by the group of Mordes and Blankenhorn (61). Of note, diabetes-susceptible rat strains have a T1D risk MHC haplotype (RT1B/D^u^), which lacks a negatively charged amino acid residue at the beta chain 57 position and is homologous to HLA-DQ8 (61). In addition to the HLA gene locus, *Iddm14*, which contains the TCR beta chain genes, is a T1D-susceptible locus (62). The group identified a TCR Vbeta allele, *Tcrb-V13S1A1*, that is shared among T1D-susceptible rat strains but not with T1D-resistant ones (63), and demonstrated that genetic elimination of this allele or depletion of T cells expressing TCRs containing Vbeta13a (product of the *Tcrb-V13S1A1* gene) abrogates diabetes development in T1D-susceptible rats (64–66). A series of these studies elegantly linked the genetic risk with a functional mechanism in which a particular TCR motif facilitates T1D development with a specific MHC molecule.

In sum, these animal studies demonstrate the presence of preferred TCR motifs in both germline-encoded and rearranged regions to recognize particular epitope sequences, which can be reasonably explained by molecular interaction between the TCR – peptide – MHC molecule. From a view of TCR biomarker development, TCR motifs shared by antigen-specific or disease-susceptible T cells can be utilized to enrich and classify TCR clonotypes that are distinctive of T1D.

### TCR Repertoires in the Pancreas of Humans

Emerging sequencing technologies and increasing availability of human samples, in particular pancreas and peripheral immune tissues isolated from organ donors having T1D, facilitate identification of islet antigen-specific or T1D-associated T cells and TCR clonotypes (7, 67–74). In the 1990’s, two groups in Spain and Japan separately analyzed TCR repertoires in the pancreas and demonstrated clonal expansion of T cells with particular Vgene segment usage in individual patients (75, 76). Importantly, the same group in Spain demonstrated that a clonally expanding TCR in islet and pancreas samples was detected in the blood of the same individual, indicating that islet-residing TCR clonotypes are detectable in peripheral blood samples (77). More recently, Brusko and colleagues further corroborated this concept by studying a larger number of individuals using a next generation sequencing technology that allows to analyze much higher numbers of T cells (72, 78). This high resolution analysis discovered that CD8 TCR clonotypes in the pancreas and draining lymph nodes are detected in peripheral blood more frequently than those expressed by CD4 T cells and provided important insights about the depth of TCR sequencing to achieve quantitative measurement.

Another important concept is to consider commonality of TCR repertoires in the pancreas across patients. We recently determined thousands of TCR clonotypes expressed by T cells in the islets of organ donors with and without T1D (73, 74). Our analysis indicated clonal expansion in the pancreas of individual donors regardless of the disease, but also found that the frequency of TCR clonotypes shared between donors is limited. This low frequency of shared TCR clonotypes may be due to diverse HLA restrictions present in different individuals. Another reason could be the fact that T cells in the islets may not be necessarily islet-specific. Indeed, multiple studies analyzing islet T cell specificity found that over half of T cell clones and lines derived from the islets did not respond to preproinsulin and other known islet epitopes (46, 70, 71, 73, 74). However, it should be noted that collecting TCR clonotypes from a larger number of donors significantly increases the number of shared clonotypes and such large TCR repertoire information allows for identifying common motifs even when not sharing entire TCR sequences, which will be essential to precisely cluster TCRs recognizing the same epitope (see below regarding TCR clustering). Thus, continuing efforts to accumulate TCR sequence information from the target organ along with epitope identification is crucial to establish a sufficient list of TCR clonotypes that can be used for disease-associated TCR biomarkers.

### Islet Antigen-Specific TCR Clonotypes in Humans

**Figure 1:**
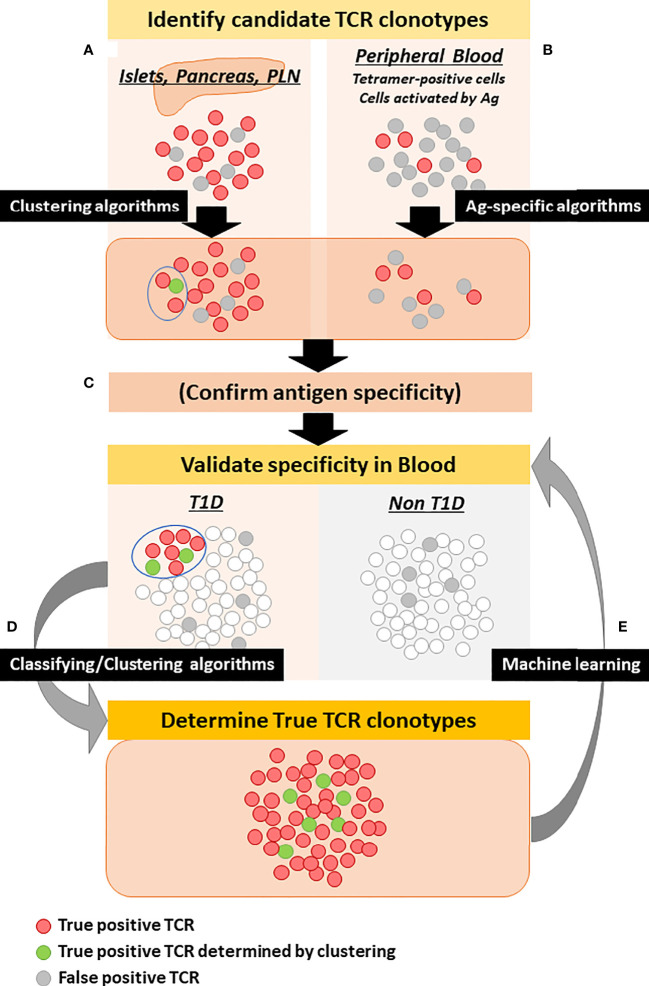
Strategy to determine disease-specific TCR clonotypes. Red and gray circles represent true and false disease-specific TCR clonotypes, respectively. Green circles are true disease-specific clonotypes determined by clustering with known disease-specific TCR clonotypes. **(A)** TCRs detected in the islets, pancreata, and pancreatic lymph nodes, in particular those for which antigen specificity has been determined as well as those that are clustered with known disease-specific TCRs, can be the initial source for disease-specific TCR candidates. **(B)** TCRs detected from peripheral blood T cells enriched by antigen stimulation or peptide-MHC-conjugated multimers are also an initial source. Antigen-specific algorithms can enrich TCR clonotypes that are truly specific to antigens. **(C)** Candidate TCR clonotypes may be assessed for specificity to islet tissues, proteins, and peptides. **(D)** Using classifying algorithms, candidate TCR clonotypes are assessed for frequency in the blood of individuals with and without T1D to determine disease specificity. Simultaneously, clustering algorithms can select additional clonotypes that are clustered with known disease-specific TCR clonotypes. **(E)** TCR clonotypes selected by classifying and clustering algorithms are used for machine learning of antigen-specific algorithms to further determine true disease-specificity.

TCRs expressed by islet-reactive T cells may be another optimal source that can be used as clonotypes for T1D biomarkers, especially if they circulate in the peripheral blood. Such clonotypes could come from T cell clones, T cell lines, hybridomas, and transductant cells that have been confirmed to respond to islet antigens, cell subsets enriched by multimer staining, and those activated or proliferated by antigen stimulation. TCR clonotypes for which reactivity to epitopes has been confirmed at a single cell level would be the most reliable source. Here we summarize islet antigen-specific TCR clonotypes that were isolated from individuals having T1D (**Table 2**). To date, over a hundred TCR alpha and beta paired sequences specific to common islet epitopes have been reported by a number of investigators, and it is notable that the majority of these TCRs were identified in the past several years (7, 45, 70, 73, 74, 79–99). However, hundreds of disease-associated TCR clonotypes are far too small to cover T1D patients having heterogeneous antigen specificity. With rapidly evolving sequencing technologies, future efforts to identify islet epitope-specific TCR clonotypes is essential to develop TCR biomarkers for T1D. In addition to TCR clonotypes listed in **Table 2**, Bonifacio and colleagues reported hundreds of TCR sequences expressed by T cells that were stained with multimer composed of islet epitopes or those proliferated in response to islet antigens (44, 45). While it is necessary to carefully validate true reactivity to antigens, this type of analysis is an excellent resource to gain T1D-associated TCR clonotypes. Computational tools to decrease the “noise” (i.e. eliminating non-specific binding TCR clonotypes) may help to enrich truly antigen-specific clonotypes (100, 101). Further, these candidate TCR clonotypes could be validated for disease specificity using larger cohorts analyzed with whole blood TCR sequencing, and then clonotypes that were detected only in individuals having various stages of T1D could be assessed for functional reactivity (**Figures 1A–C**). Retro/lentiviral transduction systems, especially in a moderate to high throughput multiplex assay, will facilitate verifying reactivity to antigens (82, 98, 102, 103).

**Table 2:** T cell receptors specific to islet epitopes.

Clone/Sequence ID	Epitope	Epitope sequence	HLA^#^	TRAV	TRAJ	CDR3a	TRBV	TRBJ	CDR3b	Source of T cells	Method to confirm reactivity	Reference
BRI4.13	GAD65:555-567	NFFRMVISNPAAT	DR4	TRAV19	TRAJ44	CALSENRGGTASKLTF	TRBV5-1	TRBJ1-1	CASSLVGGPSSEAFF	PBMC CD4	Clone/Transgenic cells	(79–81)
BRI164	GAD65:555-567	NFFRMVISNPAAT	DR4	TRAV19	TRAJ56	CALSEEGGGANSKLTF	TRBV5-1	TRBJ1-6	CASSLAGGANSPLHF	PBMC CD4	Clone/Transgenic cells	(80, 82)
T1D2-1&2	IGRP:305-324	QLYHFLQIPTHEEHLFYVLS	DR4	TRAV29	TRAJ40	CAATRTSGTYKYIF	TRBV6-6	TRBJ2-3	CASSPWGAGGTDTQYF	PBMC CD4	Clone/TCR transductant	(81)
T1D4-3&4	IGRP:305-324	KWCANPDWIHIDTTPFAGLV	DR4	TRAV2	TRAJ15	CAVEDLNQAGTALIF	TRBV5-1	TRBJ2-1	CASSLALGQGNQQFF	PBMC CD4	Clone/TCR transductant	(81)
23.G8	PPI:36-50	VEALYLVCGERGFFY	DR4	TRAV39	TRAJ56	CAWRTGANSKLTF	TRBV24-1	TRBJ2-2	CATGLAANTGELFF	Islet CD4	TCR transductant	(83)
SD52.c1	PPI:72-90	PGAGSLQPLALEGSLQKRG	DR4	TRAV4	TRAJ27	CLVGDSLNTNAGKSTF	TRBV27	TRBJ1-5	CASSWSSIGNQPQHF	PBMC CD4	Clone	(82, 84)
95.A9-1	PPI:87-101	QKRGIVEQCCTSICS	DR4.4	TRAV9-2	TRAJ18	CALRTDRGSTLGRLYF	TRBV11-2	TRBJ1-6	CASSLQSSYNSPLHF	Islet CD4	TCR transductant	(83)
Mi.1	Insulin A:1-15 (PPI: 90-104)	GIVEQCCTSICSLYQ	DR4	TRAV8-3	TRAJ44	CAVGALAGTASKLTF	TRBV29-1	TRBJ2-3	CSVEATRADTQYF	PLN CD4	Clone	(85)
Ba.14	Insulin A:1-15 (PPI: 90-104)	GIVEQCCTSICSLYQ	DR4	TRAV39	TRAJ33	CAVVNMDSNYQLIW	TRBV5-1	TRBJ2-3	CASSLATSGGGSDTQYF	PLN CD4	Clone	(85)
Ba.11	Insulin A:1-15 (PPI: 90-104)	GIVEQCCTSICSLYQ	DR4	TRAV22 TRAV26-2 ^##^	TRAJ52 TRAJ47	CADAGGTSYKLF CIPGSEEYGNKLVF	TRBV5-1	TRBJ2-3	CASSLATSGGGSDTQYF	PLN CD4	Clone	(85)
6.H11	PPI:94-108	QCCTSICSLYQLENY	DR4.2	TRAV26-1	TRAJ13	CIVRVYSGGYQKVTF	TRBV30	TRBJ2-3	CAWSARLAGGPRTQYF	Islet CD4	TCR transductant	(83)
SD32.5	PPI:94-110	QCCTSICSLYQLENYCN	DR4	TRAV26-1	TRAJ23	CIVRVSSAYYNQGGKLIF	TRBV27	TRBJ2-3	CASSPRANTDTQYF	PBMC CD4	Clone	(82, 84)
B3.3	Proinsulin:52-62 (PPI:76-86)	SLQPLALEGSL	DR4	TRAV17	TRAJ54	CATGPIQGAQKLVF	TRBV6-5	TRBJ1-1	CASSYAWGRATEAFF	PBMC CD4	Clone	(86)
K4.4/K6.4	Proinsulin:54-63 (PPI:78-87)	QPLALEGSLQ	DR4	TRAV10	TRAJ17	CVVSAKAAGNKLTF	TRBV7-8	TRBJ2-7	CASSLAGTDHYEQYF	PBMC CD4	Clone	(86)
23.F7	PPI:24-38	AFVNQHLCGSHLVEA	DR1	TRAV8-2	TRAJ29	CAVIASGNTPLVF	TRBV19	TRBJ2-3	CASKGPGTVIRADTQYF	Islet CD4	TCR transductant	(83)
55.B3	PPI:37-51	EALYLVCGERGFFYT	DR9	TRAV21	TRAJ29	CAVLPPTPLVF	TRBV18	TRBJ1-1	CASSYPGTGGARTEAFF	Islet CD4	TCR transductant	(83)
55.C10	PPI:58-72	AEDLQVGQVELGGGP	DR53	TRAV26-1	TRAJ26	CIVRSHGQNFVF	TRBV20-1	TRBJ2-7	CSARPGTRNYEQYF	Islet CD4	TCR transductant	(83)
Clone 5	Insulin B:9-23 (PPI: 33-47)	SHLVEALYLVCGERG	DQ8	TRAV21	TRAJ6	CAVKRTGGSYIPTF	TRBV11-2	TRBJ2-2	CASSSFWGSDTGELFF	PBMC CD4	Clone/TCR transductant	(82, 87, 88)
GSE.6H9	Insulin B:9-23 (PPI: 33-47)	SHLVEALYLVCGERG	DQ8, DQ8-trans	TRAV26-1	TRAJ40	CIVRVDSGTYKYIF	TRBV7-2	TRBJ2-1	CASSLTAGLASTYNEQFF	Islet CD4	TCR transductant	(73, 83)
GSE.20D11	Insulin B:9-23 (PPI: 33-47)	SHLVEALYLVCGERG	DQ8	TRAV12-3	TRAJ4	CAILSGGYNKLIF	TRBV2	TRBJ2-5	CASSAETQYF	Islet CD4	TCR transductant	(73, 83)
T1D#3 C8	Insulin B:11-23 (PPI: 35-47)^R22E^	LVEALYLVCGEEG	DQ8	TRAV17	TRAJ23	CATDAGYNQGGKLIF	TRBV5-1	TBBJ1-3	CASSAGNTIYF	PBMC CD4	Clone	(82, 89)
T1D#3 C10	Insulin B:11-23 (PPI: 35-47)^R22E^	LVEALYLVCGEEG	DQ8	TRAV12-3	TRAJ26	CATAYGQNFVF	TRBV4-1	TRBJ2-2	CASSRGGGNTGELFF	PBMC CD4	Clone	(82, 89)
19.A4	PPI:55-69	RREAEDLQVGQVELG	DQ8	TRAV8-6	TRAJ32	CAVRETGATNKLIF	TRBV20-1	TRBJ2-7	CSARPQGFSSYEQYF	Islet CD4	TCR transductant	(83)
GSE.8E3	PPI:72-87 hEL:C-peptide (HIP11)	PGAGSLQPLALEGSLQ SLQPLALEAEDLQV	DQ8, DQ8-trans	TRAV2	TRAJ37	CAVDGSGNTGKLIF	TRBV4-1	TRBJ2-7	CASSQDLAGVREQYF	Islet CD4	TCR transductant	(73, 83)
6.G4	PPI:86-100	LQKRGIVEQCCTSIC	DQ8, DQ8-trans	TRAV26-1	TRAJ8	CIVRVRNTGFQKLVF	TRBV27	TRBJ1-1	CASSPGPGNTEAFF	Islet CD4	TCR transductant	(83)
56.B1	PPI:40-54	YLVCGERGFFYTPKT	DQ2	TRAV13-1	TRAJ40	CAVLSPSGTYKYIF	TRBV7-9	TRBJ1-4	CASSLMGNPHEKLFF	Islet CD4	TCR transductant	(83)
53.A4-1	PPI:72-87	PGAGSLQPLALEGSLQ	DQ2	TRAV39	TRAJ33	CAVDPMDSNYQLIW	TRBV29-1	TRBJ2-6	CSVGTDPSGANVLTF	Islet CD4	TCR transductant	(83)
23.G6	PPI:29-43	HLCGSHLVEALYLVC	DP4	TRAV9-2	TRAJ6	CALSISGGSYIPTF	TRBV5-1	TRBJ2-5	CASSFRQGEQETQYF	Islet CD4	TCR transductant	(83, 90)
A4.13	Proinsulin:41-51 (PPI:65-75)	QVELGGGPGAG	DQ8	TRAV6	TRAJ36	CALKYGANNLFF	TRBV18	TRBJ1-1	CASSPTTGGDEAFF	Islet CD4	Clone	(70)
A1.1	Proinsulin:50-59 (PPI:74-83)	AGSLQPLALE	DQ8	TRAV25	TRAJ16	CAGGFSDGQKLLF	TRBV20-1	TRBJ2-7	CSARTEAYEQYF	Islet CD4	Clone	(70)
A1.2	Proinsulin:50-58 (PPI:74-82)	AGSLQPLAL	DQ8	TRAV20	TRAJ58	CAVIETSGSRLTF	TRBV20-1	TRBJ2-3	CSARDQQRVDTQYF	Islet CD4	Clone	(70)
A2.4	Proinsulin:52-62 (PPI:76-86)	SLQPLALEGSL	DQ8-trans	TRAV19	TRAJ49	CALSRAGTGNQFYF	TRBV5-1	TRBJ2-4	CASSLGLRGENIQYF	Islet CD4	Clone	(70)
B3.1	Proinsulin:48-59 (PPI:72-83)	PGAGSLQPLALE	DQ8	TRAV12-1	TRAJ9	CVVKSTGGFKTIF	TRBV20-1	TRBJ2-5	CSAGGLAGASQETQYF	PBMC CD4	Clone	(86)
K3.2/K9.5	Proinsulin:54-62 (PPI:78-86)	QPLALEGSL	DQ2	TRAV3	TRAJ31	CAVRGDNNARLMF	TRBV7-2	TRBJ2-2	CASSPIIWGTGELFF	PBMC CD4	Clone	(86)
K6.2	Proinsulin:49-58 (PPI:73-82)	GAGSLQPLAL	DQ8-trans	TRAV8-2/8-4	TRAJ11	CAVTPKSGYSTLTF	TRBV20-1	TRBJ2-3	CSARDLAIPDTQYF	PBMC CD4	Clone	(86)
K9.6	Proinsulin:41-51 (PPI:65-75)	QVELGGGPGAG	DQ8	TRAV26-1	TRAJ54	CIVRVEIQGAQKLVF	TRBV3-2	TRBJ2-1	CASSSPGTEYNEQFF	PBMC CD4	Clone	(86)
D1.1/D1.4	Proinsulin:34-43 (PPI:58-67)	AEDLQVGQVE	DQ8	TRAV13-1	TRAJ38	CAARNAGNNRKLIW	TRBV4-2	TRBJ2-3	CASSFRGLGGGTDTQYF	PBMC CD4	Clone	(86)
T6.1	Proinsulin:52-63 (PPI:76-87)	SLQPLALEGSLQ	DQ2, DQ2-trans	Functional alpha not detected			TRBV9	TRBJ2-1	CASSVDPGVYNEQFF	PBMC CD4	Clone	(86)
T6.6	Proinsulin:56-62 (PPI:80-86)	LALEGSL	DQ2	TRAV35	TRAJ28	CAAALSGAGSYQLTF	TRBV19	TRBJ2-3	CASRLDPSTDTQYF	PBMC CD4	Clone	(86)
T17.1	Proinsulin:56-62 (PPI:80-86)	LALEGSL	DQ2, DQ2-trans	TRAV35	TRAJ28	CAAALSGAGSYQLTF	TRBV19	TRBJ2-3	CASRLDPSTDTQYF	PBMC CD4	Clone	(86)
H3.3/H6.4	Proinsulin:52-61 (PPI:76-85)	SLQPLALEGS	DQ8-trans	TRAV19	TRAJ57	CALSGRGSEKLVF	TRBV5-1	TRBJ2-7	CASSTRTGQGGNEQYF	PBMC CD4	Clone	(86)
H3.7/H7.4/H8.5	Proinsulin:50-58 (PPI:74-82)	AGSLQPLAL	DQ8	TRAV12-1	TRAJ20	CVVNPTDDYKLSF	TRBV20-1	TRBJ2-3	CSARSLASGGPDTQYF	PBMC CD4	Clone	(86)
H11.5	Proinsulin:42-51 (PPI:66-75)	VELGGGPGAG	DQ8	TRAV26-1	TRAJ36	CIVRVVTGANNLFF	TRBV5-1	TRBJ2-5	CASSLERETQYF	PBMC CD4	Clone	(86)
E2.3	Proinsulin:54-62 (PPI:78-86)	QPLALEGSL	DQ2	TRAV30	TRAJ37	CGTEKPGSGNTGKLIF	TRBV20-1	TRBJ1-4	CSARDGARGEKLFF	PBMC CD4	Clone	(86)
E2.5	Proinsulin:35-46 (PPI:59-70)	EDLQVGQVELGG	DQ8	TRAV12-3	TRAJ5	CVISPPGRRALTF	TRBV5-4	TRBJ2-2	CASSSGTSAGTGELFF	PBMC CD4	Clone	(86)
A3.10	hEGGG : IAPP2 (HIP6)	GQVELGGGNAVEVLK	DQ8	TRAV38-1	TRAJ54	CAFFGQGAGKLVF	TRBV5-1	TRBJ2-3	CASSLSASGGATDTQYF	Islet CD4	Clone	(70, 91, 92)
A1.9	Proinsulin:42-50 (PPI:66-74) hEGGG : IAPP2 (HIP6)	VELGGGPGA GQVELGGGNAVEVLK	DQ8	TRAV20	TRAJ7	CAVQAGGNNRLAF	TRBV5-1	TRBJ1-2	CASSLERDGYTF	Islet CD4	Clone	(70, 92)
A6.15/A5.8	Proinsulin:42-50 (PPI:66-74) hEGGG : IAPP2 (HIP6)	VELGGGPGA GQVELGGGNAVEVLK	DQ8	TRAV26-1	TRAJ21	CIAIYNFNKFYF	TRBV5-1	TRBJ1-6	CASSLEASSYNSPLHF	Islet CD4	Clone	(70, 92)
A2.13	Proinsulin:42-50 (PPI:66-74) hEGGG : IAPP2 (HIP6)	VELGGGPGA GQVELGGGNAVEVLK	DQ8	TRAV26-1	TRAJ39	CIVSHNAGNMLTF	TRBV5-1	TRBJ2-5	CASSLERETQYF	Islet CD4	Clone	(70, 92)
A5.5	Proinsulin:42-50 (PPI:66-74) hEGGG : IAPP2 (HIP6)	VELGGGPGA GQVELGGGNAVEVLK	DQ8	TRAV26-1	TRAJ54	CIVRVEIQGAQKLVF	TRBV5-1	TRBJ2-5	CASSLGPGQRETQYF	Islet CD4	Clone	(70, 92)
A2.11	hEGGG : IAPP2 (HIP6)	GQVELGGGNAVEVLK	Not reported	TRAV38-1	TRAJ54	CAFMGAGAQKLVF	TRBV4-3	TRBJ2-3	CASSQILRGGPPDTQYF	Islet CD4	Clone	(70, 91)
HIP14-G10/D3	hEL : IAPP2 (HIP14)	SLQPLALNAVEVLK	DR	TRAV16 TRAV5 ^##^	TRAJ37 TRAJ40	CARSHGSGNTGKLIF CAESIASGTYKYIF	TRBV27	TRBJ2-5	CASSSGYGGETQYF	PBMC CD4	Clone	(93)
E2b	hEL:C-peptide (HIP11)	SLQPLALEAEDLQV	DQ2	TRAV8-4	TRAJ43	CAVGATNNNDMRF	TRBV5-4	TRBJ2-1	CASSPIGASGGNEQFF	PBMC CD4	Clone	(94)
ET650-2	Proinsulin:42-50 (PPI:66-74) hEGGG : IAPP2 (HIP6) HIPL11C	VELGGGPGA GQVELGGGNAVEVLK GQVELGGGNAVEVCK	DQ8	TRAV26-1	TRAJ39	CIVRVGYNAGNMLTF	TRBV20-1	TRBJ1-5	CSAIAGPNQPQHF	PBMC CD4	Clone	(92)
ET650-4	Proinsulin:42-50 (PPI:66-74) hEGGG : IAPP2 (HIP6) HIPL11C	VELGGGPGA GQVELGGGNAVEVLK GQVELGGGNAVEVCK	DQ8	TRAV26-1	TRAJ42	CIVRVAIEGSQGNLIF	TRBV5-1	TRBJ1-3	CASSLRRGDTIYF	PBMC CD4	Clone	(92)
ET650-5	hEGGG : IAPP2 (HIP6) HIPL11C	GQVELGGGNAVEVLK GQVELGGGNAVEVCK	DQ8	TRAV26-1	TRAJ9	CIVRLQSGGFKTIF	TRBV20-1	TRBJ1-2	CSAYSPGDRDFSNYGYTF	PBMC CD4	Clone	(92)
ET672-1	Proinsulin:42-50 (PPI:66-74) hEGGG : IAPP2 (HIP6) HIPL11C	VELGGGPGA GQVELGGGNAVEVLK GQVELGGGNAVEVCK	DQ8	TRAV12-2	TRAJ48	CAVNHGNEKLTF	TRBV18	TRBJ1-1	CASSPWEGRMDTEAFF	PBMC CD4	Clone	(92)
1E6	PPI:15-24	ALWGPDPAAA	A*02:01	TRAV12-3	TRAJ12	CAMRGDSSYKLIF	TRBV12-4	TRBJ2-4	CASSLWEKLAKNIQYF	PBMC CD8	Clone	(82, 95)
1D5/1D10/2B3/4C6/3E7	PPI:3-11	LWMRLLPLL	A*24:02	TRAV5	TRAJ37	CAEPSGNTGKLIF	TRBV7–9	TRBJ2-7	CASSLHHEQYF	PBMC CD8	Clone	(96)
Clone 7	IGRP:265-273	VLFGLGFAI	A*02:01	TRAV41	TRAJ48	CAVTSNFGNEKLTF	TRBV6-2/6-3	TRBJ2-7	CASSSRFVGEGLFRYGYEQYF	PBMC CD8	Clone/Transgenic cells	(97, 98)
Clone 32	IGRP:265-273	VLFGLGFAI	A*02:01	TRAV12-1	TRAJ48	CVVNILSNFGNEKLTF	TRBV20-1	TRBJ2-1	CSASRQGWVNEQFF	PBMC CD8	Clone/Transgenic cells	(97, 98)
Clone 16/17	IGRP:265-273	VLFGLGFAI	A*02:01	TRAV25	TRAJ53	CAGLGDSGGSNYKLTF	TRBV3-1	TRBJ2-4	CASSQDRWDVMSKNIQYF	PBMC CD8	Clone	(45)
Clone 22/27	IGRP:265-273	VLFGLGFAI	A*02:01	TRAV29/DV5	TRAJ53	CAASGGSNYKLTF	TRBV10-3	TRBJ1-2	CAISDRFMREGMTYGYTF	PBMC CD8	Clone	(45)
Clone#1	INS-DRiP:1-9	MLYQHLLPL	A*02:01	TRAV12-2	TRAJ34	CAVNKTDKLIF	TRBV6-1	TRBJ1-2	CASSVTGNGYTF	PBMC CD8	Clone	(99)
Clone#2	INS-DRiP:1-9	MLYQHLLPL	A*02:01	TRAV10	TRAJ8	CVVNMNTGFQKLVF	TRBV12-3/12-4	TRBJ2-2	CASSPPQGGNTGELFF	PBMC CD8	Clone	(99)
1.C1	INS-DRiP:1-9	MLYQHLLPL	A*02:01	TRAV12-1	TRAJ39	CGENNAGNMLTF	TRBV27	TRBJ2-5	CASSLQPPGTSTETQYF	Islet CD8	TCR transductant	(74)
96.A9	INS-DRiP:1-9	MLYQHLLPL	B*08:01	TRAV12-2	TRAJ39	CAVNVYNAGNMLTF	TRBV30	TRBJ1-1	CAWSVRGGSYMNTEAFF	Islet CD8	TCR transductant	(74)
D222D Clones 2	ZNT8:186-194	VAANIVLTV	A*02:01	TRAV17	TRAJ36	CAVTGANNLFF	TRBV19	TRBJ2-2	CASSIEGPTGELF	PBMC CD8	Clone	(7)
D010R clone 1E2	ZNT8:186-194	VAANIVLTV	A*02:01	TRAV35	TRAJ36	CAGTRNNLFF	TRBV19	TRBJ2-7	CASGGSSYEQYF	PBMC CD8	Clone	(7)
D010R clone 1D3	ZNT8:186-194	VAANIVLTV	A*02:01	TRAV25	TRAJ20	CAGGSNDYKLSF	TRBV6-1	TRBJ2-3	CASSSVGVDTQYF	PBMC CD8	Clone	(7)
D267T 33B8	ZNT8:186-194	VAANIVLTV	A*02:01	TRAV19	TRAJ23	CALSEATYNQGGKLIF	TRBV19	TRBJ1-3	CASSIFPNPGNTIYF	PBMC CD8	Clone	(7)
D349D 178B9	ZNT8:186-194	VAANIVLTV	A*02:01	TRAV14/DV4	TRAJ9	CAMREGLTGGFKTIF	TRBV11-2	TRBJ1-1	CASSPFLTGSNTEAFF	PBMC CD8	Clone	(7)
D351D 188D3	ZNT8:186-194	VAANIVLTV	A*02:01	TRAV19	TRAJ20	CALSPAETSDYKLSF	TRBV19	TRBJ1-1	CASTLTGFAEAFF	PBMC CD8	Clone	(7)
23.F9	PPI:1-11	MALWMRLLPLL	C*03:04	TRAV12-3	TRAJ48	CAMSALGNFGNEKLTF	TRBV19	TRBJ2-1	CASSIAGGNEQFF	Islet CD8	TCR transductant	(74)
19.A1	PPI:1-11	MALWMRLLPLL	C*03:04	TRAV8-4	TRAJ11	CAVSDQGSGYSTLTF	TRBV28	TRBJ1-5	CASSWTANQPQHF	Islet CD8	TCR transductant	(74)
20.E5	PPI:1-11	MALWMRLLPLL	C*03:04	TRAV14/DV4	TRAJ52	CAMSNAGGTSYGKLTF	TRBV28	TRBJ1-4	CASSLARYNEKLFF	Islet CD8	TCR transductant	(74)
20.F1	PPI:1-11	MALWMRLLPLL	C*03:04	TRAV14/DV4	TRAJ43	CAMRLHNNNDMRF	TRBV28	TRBJ1-5	CASIASRYNQPQHF	Islet CD8	TCR transductant	(74)
22.A10	PPI:1-11	MALWMRLLPLL	C*03:04	TRAV8-1	TRAJ13	CAVNAAGGYQKVTF	TRBV28	TRBJ2-1	CASIPDRYNEQFF	Islet CD8	TCR transductant	(74)
1.C8	PPI:1-11/2-12/2-10	MALWMRLLPLL ALWMRLLPLLA ALWMRLLPL	A*02:01	TRAV24	TRAJ58	CAFKRETSGSRLTF	TRBV13	TRBJ2-1	CASSTRLAGDEQFF	Islet CD8	TCR transductant	(74)
1.F3	PPI:2-12	ALWMRLLPLLA	A*02:01	TRAV39	TRAJ39	CAVENAGNMLTF	TRBV10-2	TRBJ2-1	CASWTVSYNEQFF	Islet CD8	TCR transductant	(74)
96.F5	PPI:3-11	LWMRLLPLL	A*02:01	TRAV8-6	TRAJ48	CAVSDISNFGNEKLTF	TRBV9	TRBJ2-3	CASSVVGLGTDTQYF	Islet CD8	TCR transductant	(74)
23.H5	PPI:3-13	LWMRLLPLLAL	A*02:01	TRAV38-2/DV8	TRAJ22	CAYRSPARQLTF	TRBV6-1	TRBJ2-7	CASSEGWGVPSYEQYF	Islet CD8	TCR transductant	(74)
1.B10-1	PPI:15-23	ALWGPDPAA	A*02:01	TRAV8-3	TRAJ33	CAVVADSNYQLIW	TRBV4-2/4-3	TRBJ2-2	CASSQTKGTGELFF	Islet CD8	TCR transductant	(74)
1.F1	PPI:15-24	ALWGPDPAAA	A*02:01	TRAV39	TRAJ41	CAVSNSGYALNF	TRBV29-1	TRBJ2-5	CSVFHRGETQYF	Islet CD8	TCR transductant	(74)
23.C12	PPI:15-24/15-25	ALWGPDPAAA ALWGPDPAAAF	A*02:01	TRAV41	TRAJ42	CAVSGGSQGNLIF	TRBV28	TRBJ1-2	CASSPPTGWGGYTF	Islet CD8	TCR transductant	(74)
93.D1	PPI:15-25	ALWGPDPAAAF	A*02:01	TRAV5	TRAJ8	CAVTKDTGFQKLVF	TRBV20-1	TRBJ2-1	CSARDHFGGSGYEQFF	Islet CD8	TCR transductant	(74)
10.C6-1	PPI:23-32	AAFVNQHLCG	C*12:03	TRAV19	TRAJ39	CALSGALNNAGNMLTF	TRBV27	TRBJ2-5	CASSLFGYRQETQYF	Islet CD8	TCR transductant	(74)
28.D3	PPI:31-41/34-41	CGSHLVEALYL HLVEALYL	A*02:01	TRAV26-2	TRAJ26	CILTDNYGQNFVF	TRBV27	TRBJ1-1	CASSLIGLNTEAFF	Islet CD8	TCR transductant	(74)
28.E6	PPI:46-54/47-54	RGFFYTPKT GFFYTPKT	A*29:02	TRAV19	TRAJ28	CALSEAGAGSYQLTF	TRBV2	TRBJ2-5	CASSPSGTSSQETQYF	Islet CD8	TCR transductant	(74)
20.G1	PPI:69-77/69-79	GGGPGAGSL GGGPGAGSLQP	C*03:04	TRAV1-2	TRAJ8	CAVRMNTGFQKLVF	TRBV9	TRBJ2-1	CASSVGMDPGLGYNEQFF	Islet CD8	TCR transductant	(74)
96.B4	PPI:91-99	IVEQCCTSI	C*05:01	TRAV12-2	TRAJ31	CAVNNARLMF	TRBV6-5	TRBJ2-1	CASRPTSGGYNEQFF	Islet CD8	TCR transductant	(74)
86.C1	PPI:91-100/92-100/92-102	IVEQCCTSIC VEQCCTSIC VEQCCTSICSL	B*41:02	TRAV19	TRAJ16	CALSEAGFSDGQKLLF	TRBV19	TRBJ2-1	CASSIQFSYNEQFF	Islet CD8	TCR transductant	(74)
84.D9	PPI:91-100/92-100/92-102	IVEQCCTSIC VEQCCTSIC VEQCCTSICSL	B*41:02	TRAV29/DV5	TRAJ43	CAASNSNDMRF	TRBV7-9	TRBJ2-1	CASSLAQREQFF	Islet CD8	TCR transductant	(74)
28.E11	PPI:91-100	IVEQCCTSIC	B*18:01	TRAV12-2	TRAJ49	CAVSMNTGNQFYF	TRBV29-1	TRBJ2-1	CSVQVYNEQFF	Islet CD8	TCR transductant	(74)
1.E9-1	PPI:92-99	VEQCCTSI	B*50:01	TRAV12-2	TRAJ34	CAVNIRYNTDKLIF	TRBV6-2/6-3	TRBJ1-5	CASSSIQGSGSGQPQHF	Islet CD8	TCR transductant	(74)
86.G3-2	PPI:94-102	QCCTSICSL	B*35:01	TRAV8-6	TRAJ33	CAVSDGYQLIW	TRBV6-1	TRBJ2-7	CASSGREAPYEQYF	Islet CD8	TCR transductant	(74)
54.F1	PPI:96-103	CTSICSLY	A*01:01	TRAV3	TRAJ26	CAVPDNYGQNFVF	TRBV7-2	TRBJ2-2	CASSLVVELFF	Islet CD8	TCR transductant	(74)

^#^HLA class II alleles: DR4 (DRB1*04:01); DR4.4 (DRB1*04:04); DR4.2 (DRB1*04:02); DR1 (DRB1*01:01); DR9 (DRB1*09:01); DR53 (DRB4*01:01); DQ8 (DQA1*03:01-DQB1*03:02); DQ8-trans (DQA1*05:01-DQB1*03:02); DQ2 (DQA1*05:01-DQB1*02:01/02:02), DQ2-trans (DQA1*03:01-DQB1*02:01); DP4 (DPA1*01:03-DPB1*04:01).

^##^Two in-frame alpha chains detected. Functional alpha not determined.

### Identification of Disease-Specific TCR Clonotypes Using Big Data

Big data analysis, which seeks to classify TCR repertoires in a specific condition using a large number of TCR samples, is an emerging strategy to identify disease-associated TCR clonotypes. A major advantage of this approach is the capability to identify disease-associated TCR clonotypes without knowing antigen specificity, thereby allowing one to include TCRs that are potentially disease-associated but not islet-specific and also those having low affinity to antigens. Indeed, specificities of large proportions of T cells in the islets are unknown (46, 70–74). Virus infections such as enterovirus and coxsackie B virus (CVB) are suggested to be involved in T1D development (104–106), and TCRs specific to these viruses could be identified by big data analysis by comparing TCR repertoires of individuals having or not having different stages of T1D. Although it has been demonstrated in infectious diseases that big data analyses can identify pathogen-specific TCR clonotypes, it has not yet been successful at identifying T1D-associated TCR clonotypes using PBMC samples from individuals with or without different stages of T1D. This could be explained by several possibilities: (1) the frequency of T1D-associated T cells may be lower than that of pathogen-specific T cells; (2) antigens involved in T1D pathogenesis, especially those at different stages of T1D, may be more heterogeneous than those in infectious diseases; (3) autoreactive TCRs could be more private (i.e. not common between patients) than those of conventional T cells; and (4) sample sizes studied to date have not been large enough. However, having large TCR data sets produced by next generation sequencing will enable machine learning algorithms to cluster and classify TCR clonotypes. Using these newly developed techniques, even infrequent disease-specific TCRs having less publicity (i.e. commonality) between people may be identified from relatively small numbers of samples. Indeed, some computational TCR classifying methods are now capable of identifying cancer patients responding to immune checkpoint inhibitors (40), and also early stages of cancer can be differentiated from healthy individuals using this type of technique (107, 108). In the next section, we will discuss how to take advantages of the latest TCR clustering/classifying techniques for T1D TCR biomarkers.

### Clustering and Classification of TCR Clonotypes

TCR clonotypes recognizing the same peptide-MHC complex often share similar motifs and features. For example, influenza-specific TCRs prefer to use TRAV38-1/TRAJ52/TRBV19/TRBJ1-2 (109–111), and melanoma (MART-1)-specific TCRs often contain an alpha chain with TRAV12-2 (112). Likewise, several features common for islet antigen-specific TCRs have been reported. We discovered that insulin B-chain-specific TCRs tend to use TRAV38-1/38-2 and other Valpha segments having similar motifs in the CDR1 and CDR2 regions (113). Also, it has been shown that a specific motif “SGGSNYKLTF” is contained in the CDR3 region of alpha chains specific to an IGRP peptide (45). More recently, crystal structure analysis of TCRs specific to a hybrid insulin peptide composed of proinsulin and islet amyloid polypeptide (IAPP) demonstrated that motifs in the TRBV5-1 segment commonly interact with amino acid residues in IAPP (92). Our work also indicates that T cell responses to hybrid insulin peptides precede clinical T1D onset (114), making these TCR clonotypes excellent candidates for biomarkers. Thus, autoreactive TCRs share commonalities and similarities, which provide clues to cluster TCRs and stratify those specific to a certain condition.

A number of algorithms to cluster or classify TCR clonotypes have been developed. Each algorithm has advantages and disadvantages as reviewed by others (115, 116), but in respect to TCR biomarker development for T1D, the algorithms can be divided to two groups. First, those that clusters TCRs by assessing similarities of TCR sequences with each other in datasets. Second, those that seek to classify TCRs by identifying similar to known antigen-specific or disease-specific TCR clonotypes. The former algorithms such as TCRdist (111), GLIPH/GLIPH2 (117, 118), ClusTCR (119), and GIANA (108) do not need information about T1D-specific epitopes and TCR sequences beforehand, and thus can be used to predict disease-specific TCR clonotypes that are specifically detected in T1D patients but not in non-diabetic subjects. On the other hand, machine learning-based algorithms that assess similarities to known antigen-specific TCR datasets to predict epitopes, such as DeepTCR (101), DeepCAT (107), TCRmatch (120), and TCRAI (100) need prior information about disease-specific TCR sequences. These algorithms show excellent performance when classifying TCRs specific to the same epitopes that were used to develop the machine learning algorithm but not for those having different specificities. Therefore, large sets of disease-specific TCR sequence information for machine training are necessary to achieve high specificity and sensitivity. Typically these types of algorithms show better performance to detect antigen-specific TCR clonotypes than the clustering-based algorithms, thereby being useful to validate TCR clonotypes once epitopes or disease-specificity are determined. Alternatively, they can be also used to ‘clean up’ (i.e. eliminate non-specific TCR clonotypes) TCR datasets that are obtained from multimer-stained T cells or those activated by antigen stimulation (**Figure 1B**).

In any case, it is essential to prepare TCR datasets from a large number of individuals with and without T1D at multiple time points to elicit the best performance by machine learning and clustering algorithms. Typically, diverse datasets rather than large data but from a limited number of samples improve learning efficiency (100). In addition, it is also important to prepare accurate TCR clonotype information to differentiate T1D patients from healthy subjects. There are now several TCR databases available, which accumulate and curate information about TCR sequences along with target peptide-MHC complexes, such as VDJbase (121, 122), IEDB (123), VDJdb (124), iReceptor (125), and McPAS-TCR (126). While these are incredibly useful resources, a proportion of islet-specific clonotypes is still very small, accounting for only ~100 out of tens of thousands of clonotypes, the majority of which are specific to viruses and tumor antigens. Assuming that self-reactive TCR clonotypes are more heterogeneous and rarer compared to pathogen-specific ones, there is a need for higher numbers of clonotypes specific to T1D. Thus, identifying a large set of accurate disease-specific TCR clonotypes will be a key component to achieve successful big data analysis, which will ultimately lead us to establish TCR biomarkers in T1D (**Figure 1**).

## Perspective

**Figure 2:**
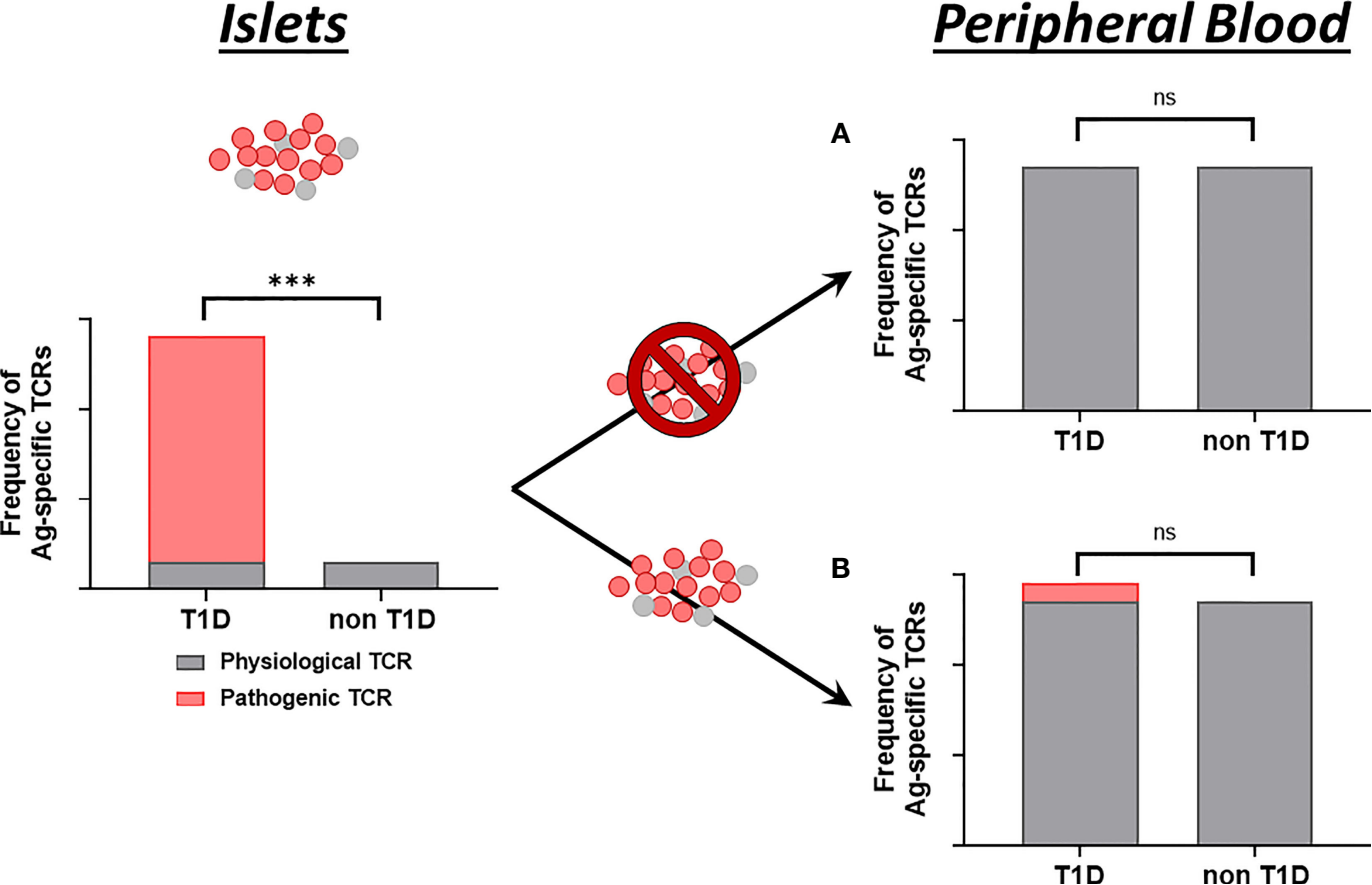
Proposed models of islet-specific T cell detection in the blood. While pancreatic islets contain a certain amount of T cells regardless of disease status (gray bars), only islets of individuals with T1D contain T cells that are highly reactive to islet antigens (red bar). Model **(A)** Leak of T cells from the islets to peripheral blood is limited. T cell repertoires specific to islet antigens are not different between individuals with and without T1D. Model **(B)** There are substantial amounts of islet-specific but not disease-specific T cells in the blood regardless of disease status (gray bars). T cells in the islets do circulate in the blood (red bar), but the total numbers of islet-specific T cells are not different between individuals with and without T1D. Enumerating only T cells derived from the islets can identify individuals having T1D. TCR clonotypes are a distinct property to identify islet-derived T cells. *** significantly different. ns, not significant.

It is still controversial whether T1D patients have distinct islet antigen-specific T cell subsets in the blood compared to healthy individuals. Even in the pancreas, non-diabetic organ donors have preproinsulin-specific T cells in the exocrine compartment, but such antigen-specific T cells accumulate into the islets over the course of T1D progression (127). In the islets, we recently demonstrated that only T1D donors have CD8 T cells highly reactive to preproinsulin (74). Mallone and colleagues also reported that pancreata of T1D donors have a higher number of zinc transporter-8-specific T cells than non-diabetic controls (7). Thus, multiple studies demonstrate that islets of T1D individuals have distinct T cell repertoires from those without diabetes. However, a number of studies indicate that healthy individuals have islet-antigen specific T cells in the blood (7, 113, 128–131), and depending on cell subsets examined, some studies including those looking into pathogenic T cells show that T1D patients have higher numbers of islet-specific T cells, whereas others do not detect differential islet-specific T cells in T1D patients. This controversy could be explained by either (1) detectable numbers of pathogenic T cells in the islets do not leak into the peripheral blood (**Figure 2A**); or (2) pathogenic T cells in the islets do indeed circulate, but because there are already a number of islet-specific (but not harmful) T cells in the circulation, the total numbers of islet-specific T cells (i.e. pathogenic T cells leaked from the islets plus non-pathogenic T cells) are not differentiated enough in the blood of T1D patients from healthy individuals (**Figure 2B**). Given evidence that a portion of T cell repertoires are shared between pancreas, pancreatic lymph nodes, and peripheral blood cells (72), and that TCR repertoires in the islets of T1D organ donors are clonally distinct from those of non-diabetic donors (74), if the latter hypothesis (**Figure 2B**) is correct, islet-derived TCR sequences will be a powerful marker to discriminate pathogenic from physiological T cells, thereby capable of stratifying individuals with active insulitis prior to clinical T1D onset.

To develop practical TCR biomarkers in T1D, a number of obstacles need to be overcome, some of which may be unique to autoimmune diseases. These challenges can be considered from the view of (1) publicity, (2) abundancy, and (3) disease-specificity.

1. Publicity
  It will be important to understand the frequencies of public *vs* private TCR clonotypes that are specific to the T1D disease state, and these likely fluctuate over time during T1D development. Given the genetic risk associated with HLA class II genes, heterogeneity provided by HLA diversity could be smaller than other diseases for TCR clonotypes expressed by CD4 T cells. However, autoreactive T cells, which often bind to peptide-MHC complexes with low affinity, may have a larger TCR repertoire than conventional anti-pathogen T cells, resulting in less commonality. Therefore, frequency of public T1D-specific TCR clonotypes may be low. Strategies that compare TCR repertoires in each individual such as pre and post treatment (40) do not need to consider publicity of clonotypes, and therefore may be more easily applicable to T1D immune intervention studies.
2. Abundancy
  Theoretically, 10^15^-10^16^ diverse TCR clonotypes can be assembled (12–14); however, a practical TCR repertoire size is estimated to be about 10^8^-10^10^ per person (15, 17, 18). This indicates that the frequency of target clonotypes is extremely low. However, there is evidence that identical clonotypes are persistently detected from the same individuals over time (44, 81, 93, 132). We believe quantitative resolution of TCRs will need to be increased. This could be achieved by enriching samples before sequencing (e.g. beads enrichment by antigen-specific multimers). Another very attractive approach is to target sequencing to TCRs containing a preferred Vgene segment of interest, thus greatly enhancing the depth of sequencing by analyzing clonotypes that can be obtained for a specific V allele. Blood sample volume needed to quantitatively evaluate frequency of disease-associated TCR clonotypes is another important consideration, which will need to be addressed given that the T1D disease process does begin in young children.
3. Disease-Specificity
  Identification of disease-specific TCR clonotypes is an essential component to develop robust T1D TCR biomarkers. A larger number of TCR clonotypes with higher specificity to the disease that are in place will allow for more sensitive and specific assays. Therefore, the key is how to select such truly disease-specific TCR clonotypes. As illustrated in **Figure 1**, both accumulation of actual TCR datasets produced from individuals with and without T1D and computational big data analysis will facilitate the development of biomarkers. While the majority of TCR big data analysis currently uses only CDR3-beta sequences, it has been demonstrated that inclusion of entire sequence information such as V and J segments, in particular CDR1 and CDR2 sequences, increases accuracy of classifying TCR clonotypes (100, 120). While the number of T1D-specific clonotypes that have been determined so far is low, evolutions in both TCR sequencing technologies and computational analysis strategies will dramatically impact this effort.

In conclusion, the antigen receptor on disease specific T cells holds promise for a non-cell based biomarker of not only the presence of T1D but disease activity as well. Efforts to define the TCR repertoire within the human pancreas of T1D and non-T1D organ donors is underway with a need to define the antigen specificity and HLA restriction of these identified clonotypes. Those clonotypes that are shared between individuals with T1D, frequent, and circulate from the pancreas and pancreatic lymph nodes to the peripheral blood are prime candidates for deep sequencing and clustering of TCRs using developed computational analyses.

## Author Contributions

MN and AM wrote and edited the manuscript. All authors contributed to the article and approved the submitted version.

## Funding

This work was supported by the National Institutes of Diabetes and Digestive and Kidney Diseases (R01DK099317, R01DK032083, R01DK108868, DP3DK110845, P30DK116073, Juvenile Diabetes Research Foundation (2018-480-S-B, 2020-911-A-N, 2018-557-Q-R), the Leona M. & Harry B. Helmsley Charitable Trust (2103-05093), and the Culshaw Family Junior Investigator Award.

## Conflict of Interest

The authors declare that the research was conducted in the absence of any commercial or financial relationships that could be construed as a potential conflict of interest.

## Publisher’s Note

All claims expressed in this article are solely those of the authors and do not necessarily represent those of their affiliated organizations, or those of the publisher, the editors and the reviewers. Any product that may be evaluated in this article, or claim that may be made by its manufacturer, is not guaranteed or endorsed by the publisher.
